# Weapons Make the Man (Larger): Formidability Is Represented as Size and Strength in Humans

**DOI:** 10.1371/journal.pone.0032751

**Published:** 2012-04-11

**Authors:** Daniel M. T. Fessler, Colin Holbrook, Jeffrey K. Snyder

**Affiliations:** Department of Anthropology and Center for Behavior, Evolution, and Culture, University of California Los Angeles, Los Angeles, California, United States of America; Faculty of Medicine University of Leipzig, Germany

## Abstract

In order to determine how to act in situations of potential agonistic conflict, individuals must assess multiple features of a prospective foe that contribute to the foe's resource-holding potential, or formidability. Across diverse species, physical size and strength are key determinants of formidability, and the same is often true for humans. However, in many species, formidability is also influenced by other factors, such as sex, coalitional size, and, in humans, access to weaponry. Decision-making involving assessments of multiple features is enhanced by the use of a single summary variable that encapsulates the contributions of these features. Given both a) the phylogenetic antiquity of the importance of size and strength as determinants of formidability, and b) redundant experiences during development that underscore the contributions of size and strength to formidability, we hypothesize that size and strength constitute the conceptual dimensions of a representation used to summarize multiple diverse determinants of a prospective foe's formidability. Here, we test this hypothesis in humans by examining the effects of a potential foe's access to weaponry on estimations of that individual's size and strength. We demonstrate that knowing that an individual possesses a gun or a large kitchen knife leads observers to conceptualize him as taller, and generally larger and more muscular, than individuals who possess only tools or similarly mundane objects. We also document that such patterns are not explicable in terms of any actual correlation between gun ownership and physical size, nor can they be explained in terms of cultural schemas or other background knowledge linking particular objects to individuals of particular size and strength. These findings pave the way for a fuller understanding of the evolution of the cognitive systems whereby humans – and likely many other social vertebrates – navigate social hierarchies.

## Introduction

Violent conflict with conspecifics is a fundamental factor influencing fitness in many social species, humans included. We can therefore expect that such species will possess adaptations that facilitate decision-making in potentially agonistic interactions, as the individual must determine whether is it best to fight, flee, or appease the prospective foe. The likelihood that the actor will prevail without incurring unsustainable costs is the product of many features of the actor and the foe, as attributes such as the potential combatants' relative size, strength, sex, age, health, and number of allies all play a role; in humans, access to weapons adds to the complexity of this calculation. Importantly, when multiple factors must be weighed, decision-making can be expedited by compiling said factors into a single summary representation. Here, we explore the hypothesis that, in situations of potential agonistic conflict, such a variable takes the form of a representation wherein conceptualized size summarizes the assessed resource-holding potential, or formidability, of the foe relative to that of the actor.

Across diverse species, physical size and, relatedly, strength, are elementary determinants of formidability, and this is also true of humans [1]. The deep antiquity of the contributions of size and strength to formidability raises the possibility that, as species evolve more complex behavioral repertoires, with corresponding increases in the range of factors influencing formidability, size and strength may come to be employed as the core dimensions of a cognitive representation that summarizes diverse determinants of relative formidability, such that the greater the foe's formidability relative to that of the actor, the larger and stronger the foe is conceptualized as being, even when the foe's formidability does not derive from actual physical size or strength. Note that, while the phylogenetic thesis holds that the postulated system whereby relative formidability is represented is innate, in species capable of complex behavior, understanding the diverse determinants of formidability will often be partially or wholly dependent on learning – innate systems can process, and even rely on, learned input (e.g., about the lethal affordances of evolutionarily novel objects).

Bolstering the likelihood that the representational system described above exists in humans, the aforementioned phylogenetic thesis is directly paralleled by a mutually compatible ontogenetic thesis. A wide variety of cognitive representations draw on bodily experience, often without explicit recognition of the relationship between representations and their sources [2]. This suggests that representations of relative formidability may be the product of lived events. Even in peaceful societies, from infancy onward, children inevitably have the recurrent experience that conflicts are won by the bigger, stronger person. Hence, over the course of development, size and strength may come to play a central role in representations of relative formidability.

If representations of a potential foe employ conceptualized size and strength as a medium for summarizing formidability, then augmenting the foe's formidability should cause the actor's conception of the foe's size and strength to increase. In humans, weapons are a primary determinant of victory in dyadic violence, and the modern handgun is prototypic in this regard. We therefore sought to test the above prediction by exploring whether knowing that someone possesses a gun increases estimations of that person's size and strength. Before investigating this question, however, we first had to rule out a potential confound.

Sell et al. [3] documented that human physical strength is correlated with the propensity to engage in coercive behavior (see also [4]). Although the authors found no effect of height in this regard, other results [5] suggest that this too may occur (see also [6], but see also [7]). It is therefore possible that, because guns enhance coercive capacity, being more prone to employ coercion, larger people may be more likely to purchase guns. If so, then demonstrating that knowing that someone possesses a gun increases participants' estimations of the target individual's size could not be taken as evidence supporting our representational hypothesis, as participants might simply be reporting correlations that they have previously observed. We therefore conducted a preliminary study in which we surveyed gun owners to ascertain whether they are taller than those who do not own guns.

## Ethics Statement

All studies reported here were examined and approved by the University of California, Los Angeles Institutional Review Board. As per said approvals, in each study, participants were initially presented with a web-based written information sheet describing the study procedures, any potential risks or discomforts, the identity and contact information of the first author, and compensation, if any. Participants indicated their consent to participate by clicking on a web link so marked. As participation was anonymous in all studies, signed informed consent was not collected.

## Pre-study

### Participants

Three hundred and forty-four adults living in the United States were recruited in multiple U.S. cities via Craigslist.org to participate in an unpaid online study titled “Traits of Gun Owners—a 2-minute Study;" advertisements explicitly solicited participation by gun owners. Data were pre-screened for incomplete or frivolous responses (e.g., participants who stated that they had not answered truthfully, etc.), leaving a final sample of 338 adults (114 females) with a mean age of 39.28 years (*SD*=13.96). The ethnicity of the sample was 92.4% White, 1.5% Hispanic/Latin American, and 6.1% other or mixed ethnicities. 60.2% of female participants and 85.8% of male participants owned guns.

### Materials and Methods

The survey consisted of demographic questions, including items addressing gun ownership and participant height. In this and subsequent studies, participants' self-reported ethnic identities were collected to provide a rough measure of the extent to which recruitment protocols reached multiple audiences in the U.S. Also included in the Pre-study were questions concerning individual differences (e.g., political orientation) designed to explore research questions orthogonal to the present enterprise.

### Results and Discussion

#### Gun Ownership and Height

All tests of significance reported in this paper are two-tailed. ANOVAs of data segregated by gender revealed no significant difference between the heights of participants who owned firearms and those who did not (*p*s>.15).

Having determined that it is unlikely that perceptions of gun possessors as larger could be driven by real-world correlations between gun ownership and physical size, we proceeded to a series of studies in which participants were asked to judge the size and strength of target men on the basis of photographs depicting only their hands holding either a handgun or various construction tools having a pistol-like handle. Because men are more likely to own guns than are women [8], and are larger and stronger than women, in order to ensure that any perceived differences in size were not due to spurious perceived differences in the sex of the models holding the objects, in this study we selected comparison objects likely to be as strongly associated with men as are guns. The construction trades primarily employ men, hence construction tools were selected as a comparison group.

## Study 1

### Participants

Five hundred and thirty-five adults living in the United States were recruited via Craigslist.org to participate in an online study titled “Judging Height From Hands," which included a $100 Amazon.com gift certificate raffle incentive. Data were screened prior to analysis for overt suspicion regarding the hypothesis, and incomplete or frivolous responses. In particular, participants who estimated any of the men depicted in the hand photographs to be taller than 220 cm or shorter than 150 cm were excluded, as these extremes fall outside of 99.9% of the U.S. male population [9]. The final sample consisted of 424 adults (314 female) with a mean age of 34.2 years (*SD*=12.82). The ethnicity of the sample was 80.8% White, 6.2% Hispanic/Latin American, 2.2% Asian, 2.2% Black/African American, and 4.7% other or mixed ethnicities.

### Materials and Methods

**Figure 1:**
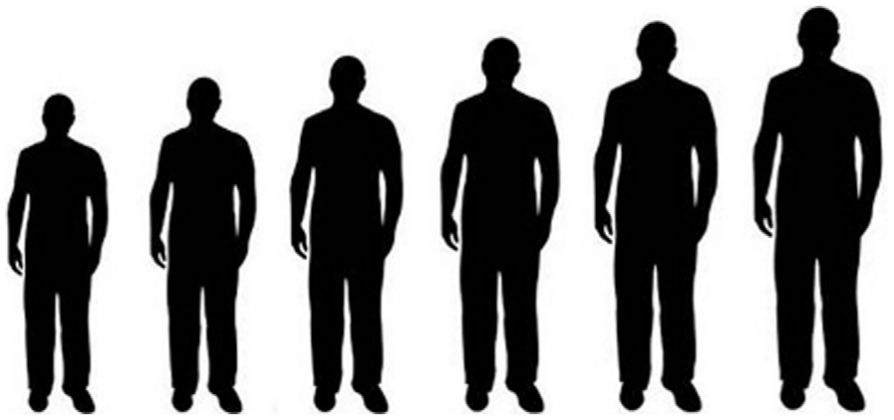
Array used by participants to provide estimates of size of target individual.

**Figure 2:**
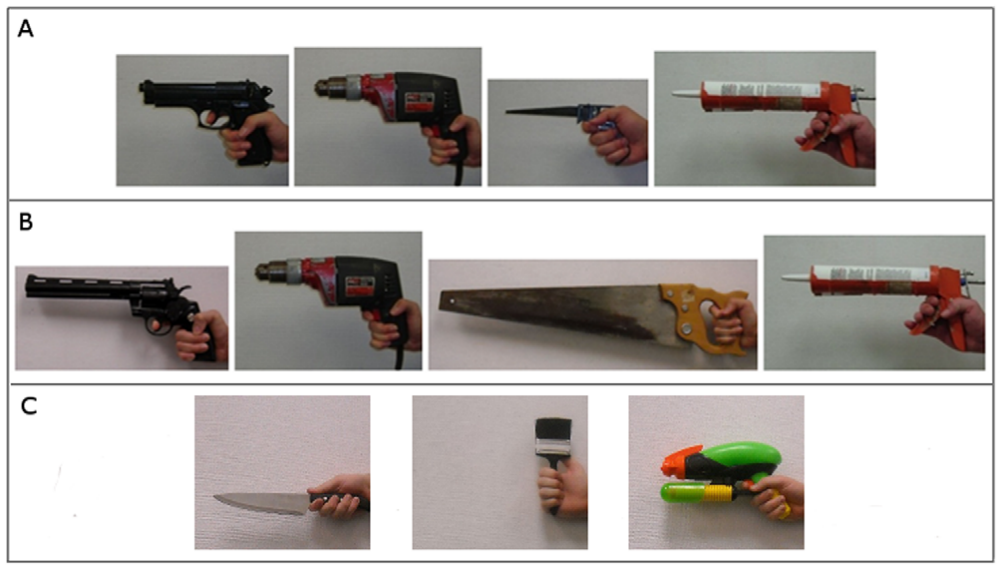
Stimuli. Panel A: In Study 1, participants rated the height and size of men holding a .45 caliber handgun, a drill, a small handsaw, and a caulking gun. Panel B: In Study 3, participants rated the height, size, and muscularity of men holding a .357 caliber handgun, a drill, a large handsaw, and a caulking gun. Panel C: In Study 5, participants rated the height, size, and muscularity of men holding a kitchen knife, a paintbrush, and a toy squirt gun. In Study 2, participants rated the potential lethality of the objects shown in Panels A and B. In Study 4, participants rated the potential lethality of the objects shown in Panel C, as well as identifying the age (adult vs. child) and gender of the persons most associated with using each object. Photographs, presented to participants in color, were resized so that the objective dimensions of each hand displayed on the participant's computer screen remained constant across all images.

The study was framed to participants as an investigation of whether hand characteristics can reveal height. In a within-subjects design, participants were asked to estimate the height (in feet and inches) and overall size (using a 6-point silhouette array; see Figure 1) of four men based on photographs of their hands holding familiar objects, ostensibly included to provide scale: a power drill, a small handsaw, a caulking gun, and a .45 caliber handgun (see Figure 2, Panel A). The photographs depicted the right hands of five White models who were selected on the basis of the similarity of their respective right hands, such that the right hands of all of the models were nearly identical in size and appearance (i.e., similar pigmentation and amount of body hair, no visible scars, tattoos, jewelry, etc.). A given object was held by a different hand model in each of five image sets. In order to minimize noise introduced by possible order effects, the sequence of images within each set was randomized, with the constraint that the handgun image was never presented first (the latter restriction was instituted in order to avoid cluing participants as to the centrality of the gun in the hypothesis being tested). Participants were randomly assigned to view one image set. Thus, one-fifth of the participants saw the handgun held by Model A, one-fifth of the participants saw the handgun held by Model B, and so on; the same was true for each of the objects depicted, and the sets of images were constructed such that, within a given set, each object was held by a different model. This design ensured that any systematic differences in participants' estimations of the size of persons as a function of the objects held could not be driven by features of the models' hands, since, when responses are pooled across participants, a given model will exercise the same effect (if any) on estimations associated with each of the four objects. Following the height and size estimations, participants were asked demographic questions, thanked, and debriefed.

### Results and Discussion

#### Effects of Object Order

MANOVAs revealed no significant effects of participant sex on estimations of height or size. Accordingly, subsequent analyses collapsed participant sex. There were significant effects of the order of image presentation on height estimations. However, these effects were minor, and were driven primarily by one item (the caulking gun). As order of presentation is orthogonal to the predictions at issue, order was therefore controlled for in subsequent analyses.

#### Height Estimates

A repeated measures ANCOVA controlling for order revealed a significant main effect of object condition on estimations of height, *F*(3, 1266)=7.14, *p*<.0001, *η* ^2^_p_=.02 (see Table 1 for means). As predicted, planned contrasts revealed that the men holding the handgun were estimated to be taller than the men holding the drill, the small handsaw, and the caulking gun (*p*s<.0001).

**Table 1:** Estimated Height and Size (Study 1).

	.45 caliber handgun	drill	small saw	caulking gun
height *Mean (SD)*	69.45 (2.79)	68.03 (2.88)	68.75 (2.82)	68.63 (2.88)
size *Mean (SD)*	4.10 (1.05)	3.54 (1.11)	3.84 (1.04)	3.74 (1.08)

*Note.* N=424. Estimated heights are in inches. The men whose hands were pictured holding the .45 caliber handgun were estimated to be both taller and larger than all of the other men (*p*s<.0001); see text for analyses.

#### Size Estimates

A repeated measures ANCOVA controlling for order revealed a significant main effect of object condition on estimations of overall size, *F*(3, 1254)=5.88, *p*<.01, *η* ^2^_p_=.01 (see Table 1 for means). As predicted, planned contrasts revealed that the men holding the handgun were estimated to be larger than the men holding the drill, the small handsaw, and the caulking gun (*p*s<.0001).

Results of Study 1 supported our prediction that an individual possessing a gun would be conceptualized as larger than individuals possessing tools. Interestingly, however, though perceived as smaller than the gun-holders, the men holding the small handsaw were nevertheless conceptualized as relatively large. Two divergent explanations suggest themselves. On the one hand, participants may view the small handsaw as having weapon-like affordances, in which case the same phenomenon may underlie perceptions of the saw-holders and the gun-holders. On the other hand, compared to most handsaws, the saw depicted is quite small, hence, if participants reference a prototypical handsaw in making their estimations, they may view the hand holding the saw, and thus the man, as quite large. In order to clarify these results, we investigated the extent to which the given objects are thought to have offensive affordances.

## Study 2

### Participants

One hundred and eight adults living in the United States were recruited via the website MechanicalTurk.com to participate in an online study titled “Your Impressions of Everyday Objects" in exchange for $0.50 compensation. Data were screened prior to analysis for incomplete or frivolous responses (e.g., rating the handgun as ‘not at all dangerous’). The final sample consisted of 102 adults (46 female), with a mean age of 34.71 years (*SD*=12.57).

### Materials and Methods

In a within-subjects design, participants were asked to rate the harmfulness of the objects used in Study 1 if employed as weapons, using a 9-point Likert scale (1=*Not at all harmful*, 5=*Moderately harmful*, 9=*Extremely lethal*). The objects were presented using images taken from Study 1, with all objects held by the same hand model; in light of the effects of the small handsaw in Study 1, an additional photo, using the same model, depicted a large handsaw. The objects were presented in fixed order: caulking gun, small handsaw, power drill, large handsaw, .45 caliber handgun. The handgun image was included as an attention check, as the lethality of firearms is not in question. Participants were then asked demographic questions, thanked, and debriefed.

### Results and Discussion

#### Effects of Sex

A MANOVA revealed significant effects of participant sex on ratings of the dangerousness of the objects; sex was therefore controlled for in subsequent analyses.

#### Perceptions of Object Danger

A repeated measures ANCOVA controlling for sex revealed a significant main effect of object condition for ratings of harmfulness, *F*(4, 400)=223.67, *p*<.000001, *η* ^2^_p_=.69 (see Table 2 for means). As predicted, planned contrasts revealed that the handgun was rated as more dangerous than all of the other objects (*p*s<.000001). In contrast, the small handsaw was not rated as significantly more or less dangerous than the drill. Lastly, the large handsaw, though far below the handgun, was nevertheless rated as significantly more dangerous than the other objects (*p*s<.01) (of potential relevance here, the latest film in the *Saw* horror movie series was heavily advertised in the U.S., and enjoying commercial success, at the time that these studies were conducted).

**Table 2:** Ratings of Object Lethality (Study 2).

	.45 caliber handgun	large saw	drill	small saw	caulking gun
lethality *Mean (SD)*	8.95 (.22)	6.75 (1.91)	6.07 (1.89)	5.75 (1.93)	3.10 (1.63)

*Note.* N=108. The .45 caliber handgun and the large handsaw were rated as potentially more lethal than all of the other objects (*p*s<.01); see text for analysis.

Having clarified that the effect of the small handsaw on perceived size found in Study 1 was likely due to the small size of the saw rather than its affordances as a weapon, we sought to replicate and extend the results of Study 1 without the confounding effects of the small handsaw. We therefore replaced the images depicting this item with images depicting a large handsaw, using the same hand models employed in Study 1. To ensure that the handgun effect found in Study 1 could not similarly be explained as owing to the comparatively large size of the hand relative to the object, we paralleled this change by replacing the images of the .45 caliber handgun with images of a (much larger) .357 magnum handgun, again using the same hand models (see Figure 2, Panel B). These changes maximized the likelihood that estimations of height or overall size would derive from perceived formidability, rather than from the relative scale effects of holding small objects. To explore our notion that representations of relative formidability employ a combination of conceptualized size and strength, we added a measure of perceptions of the muscularity of the target men. Lastly, to replicate the Pre-study, we also included questions about gun ownership and participant height.

## Study 3

### Participants

Recruitment and compensation were identical to Study 1, with the exception that the title was changed to “Judging Bodies From Hands;" 658 adults participated. Screening using the same criteria employed in Study 1 left a sample of 628 (497 female) with a mean age of 34.4 years (*SD*=12.95). The ethnicity of the sample was 83.9% White, 4.5% Hispanic/Latin American, 2.5% Asian, 2.7% Black/African American, and 6.4% other or mixed ethnicities.

### Materials and Methods

**Figure 3:**
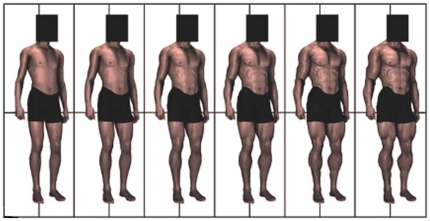
Array used by participants to provide estimates of muscularity of target individual. Modified with permission from Frederick DA, Peplau LA (2007) The UCLA Body Matrices II: Computer-generated images of men and women varying in body fat and muscularity/breast size to assess body satisfaction and preferences. 8TH Annual Meeting of the Society for Personality and Social Psychology.

The design and framing paralleled Study 1. In addition to the height and size estimation measures employed in Study 1, participants were also asked to rate perceived muscularity using a 6-point array (see Figure 3). Questions regarding gun ownership and participant height were added to the demographic items.

### Results and Discussion

#### Effects of Object Order and Sex

MANOVAs revealed significant effects of participant sex on height estimations and size ratings. These effects were minor and inconsistent across objects (compared to male participants, female participants judged the models holding the drill to be slightly taller/larger, and the models holding the caulking gun to be slightly shorter/smaller). Sex was therefore controlled for in subsequent analyses of height and size ratings. A MANOVA also revealed significant effects of the order of image presentation on height, overall size, and muscularity; as in Study 1, these effects were minor. Order of presentation was therefore controlled for in subsequent analyses.

#### Height Estimates

Repeated measures ANCOVAs with a Greenhouse-Geisser correction controlling for order and sex revealed a significant main effect of object condition for estimations of height, *F*(2.956, 1832.92)=16.73, *p*<.000001, *η* ^2^_p_=.03 (see Table 3 for means). As predicted, planned contrasts revealed that the men holding the handgun were estimated to be taller than the men holding the drill (*p*<.0001), and the men holding the caulking gun (*p*<.000001); however, this was not true with regard to the men holding the saw (*p*>.1). Consonant with the ranking of the tools in terms of lethality obtained in Study 2, the men holding the saw (the tool judged to be most lethal in Study 2) were estimated to be taller than the men holding the drill (*p*<.01) and the men holding the caulking gun (*p*<.000001).

**Table 3:** Estimated Height, Size, and Muscularity (Study 3).

	.357 caliber handgun	drill	large saw	caulking gun
height *Mean (SD)*	69.83 (2.85)	69.31 (2.54)	69.64 (2.57)	67.54 (2.45)
size *Mean (SD)*	3.94 (1.26)	3.83 (1.06)	3.89 (1.11)	3.00 (1.11)
muscularity *Mean (SD)*	2.81 (1.20)	2.75 (1.14)	2.58 (1.135)	2.39 (1.20)

*Note.* N=628. Estimated heights are in inches. The men whose hands were pictured holding the .357 caliber handgun were estimated to be both taller and larger than all of the other men (*p*s<.01); see text for analyses.

#### Size Estimates

A repeated measures ANCOVA with a Greenhouse-Geisser correction controlling for order and sex revealed a significant main effect of object condition for estimations of size, *F*(2.90, 1791.836)=24.54, *p*<.000001, *η* ^2^_p_=.04 (see Table 3 for means). As predicted, planned contrasts revealed that the men holding the handgun were estimated to be larger than the men holding the caulking gun (*p*<.000001) and the drill (*p*<.05), but, again, this was not true with regard to the saw (*p*>.3). The men holding the saw were estimated to be larger than the men holding the caulking gun (*p*<.000001) (judged to be much less lethal than the saw in Study 2), but not the men holding the drill (*p*>.2) (judged to be only slightly less lethal than the saw).

#### Muscularity Estimates

A repeated measures ANCOVA with a Greenhouse-Geisser correction controlling for order of object presentation revealed a main effect of object condition for estimations of muscularity, *F*(2.95, 1831.682)=3.46, *p*<.02, *η* ^2^_p_=.01 (see Table 3 for means). As predicted, planned contrasts revealed that the men holding the handgun were estimated to be more muscular than the men holding the caulking gun (*p*<.000001) and the saw (*p*<.0001), but (possibly reflecting knowledge of the strength required to handle a large drill), not the drill holders (*p*>.3), although the means were in the predicted direction. The men holding the saw were estimated to be more muscular than the men holding the caulking gun (*p*<.01), but less muscular than the men holding the drill (*p*<.01).

#### Gun Ownership and Height

Seventeen percent of female participants and 36.6% of male participants reported owning a gun, figures roughly similar to the U.S. national averages of 11% and 42%, respectively [8]. Replicating the results of the Pre-study, ANOVAs of data segregated by gender revealed no significant difference between the heights of participants who owned firearms and those who did not (*p*s>.35).

Study 3 bolstered the results of Study 1, as participants generally perceived gun possessors as taller than tool possessors, and the same was generally true with regard to perceptions of overall size and muscularity. Hints of the same type of patterns are evident when perceptions of tool possessors are viewed in light of the results of Study 2 regarding the perceived affordances of each tool as a weapon, as men possessing the more lethal tool were seen as taller, and, albeit less consistently, larger and more muscular, than men possessing the less lethal tool. The substantial impact of gun possession on perceptions of height found in Study 1 cannot be explained in terms of participants' use of the gun as a source of scale, as Study 3 produced the same pattern of results despite the fact that a much larger gun was displayed, making the model's hand appear proportionately smaller in the gun images in Study 3 compared to the gun images in Study 1. Lastly, the perceptual effect of gun possession is unlikely to be due to any actual correlation between gun ownership and bodily characteristics as, replicating the Pre-study, we again found that gun owners were not taller than non-owners.

Taken together, the results of Study 1 and Study 3 indicate that participants perceive individuals who possess handguns as being larger and stronger than individuals who possess less lethal construction tools. While this pattern is consistent with the hypothesis that conceptualized size and strength are used to represent the relative formidability of a potential foe, two competing explanations also exist. First, it is possible that these results reflect the influence of cultural schemas that describe the typical attributes of individuals possessing various objects. Popular entertainment is replete with depictions of large, exceptionally muscular men wielding guns. Accordingly, knowing that an individual possesses a handgun may activate well-learned schemas concerning hypertrophied macho heroes, and said schemas may then be consulted when making estimations regarding the physical attributes of the target individual. While construction tools are similarly associated with men, because mass-media portrayals of construction workers do not depict them as vastly above-average in size and muscularity, the difference between participants' estimations of gun holders and their estimations of tool holders could simply reflect prevailing cultural schemas. Second, large handguns of the types depicted in Studies 1 and 3 generate substantial recoil force. As a consequence, considerable strength is necessary if such handguns are to be handled properly, hence the patterns evident in Studies 1 and 3 could reflect inferences – whether consciously arrived at or not – based on the mechanical properties of the objects at issue. The latter explanation is consistent with the finding in Study 3 that participants perceived the men holding the large handsaw and the power drill as larger and stronger than the men holding the caulking gun, as a large handsaw and a power drill both demand greater strength than does a caulking gun.

In order to discriminate between the above explanations, employing the same basic format, we designed another study wherein the depicted object having the greatest affordances as a weapon is a kitchen knife, an implement that can be expected to be stereotypically associated with women rather than with large, muscular men. As a comparison object, we selected a paintbrush of the type used in the construction trades, a harmless object likely to be associated with men. If cultural schemas linking objects to types of persons are the primary determinant of conceptualizations of our unseen models, then participants should estimate the possessor of the paintbrush to be larger and stronger than the possessor of the kitchen knife. However, if such conceptualizations are primarily driven by the impact of the given object on the possessor's formidability, then the opposite pattern should manifest, as, when wielded as a weapon, a kitchen knife is more dangerous than a paintbrush. Note that this design simultaneously addresses the second competing explanation, namely that participants in Studies 1 and 3 assumed that individuals holding guns were larger and stronger because strength is required to properly fire a handgun, as strength is not required to properly handle a kitchen knife. Lastly, to further examine the validity of the size-by-association explanation, as a second comparison object we included a brightly-colored fantastical squirt gun, an object that is clearly a toy, and hence can be expected to be associated with boys rather than with adult men. The design thus contained a lethal object that we expected to be associated with women, a nonlethal object that we expected to be associated with men, and a nonlethal object that we expected to be associated with children.

To ensure that the expected associations of kitchen knives with women, paintbrushes with men, and squirt guns with boys are indeed widespread, and to ensure that kitchen knives are indeed viewed as having significantly greater affordances as weapons, we first conducted a background study examining the social associations and perceived lethality of a kitchen knife, a paintbrush of the type used in the construction trades, and a toy squirt gun.

## Study 4

### Participants

One hundred participants (57 female) living in the United States were recruited via the website MechanicalTurk.com to participate in a study titled “Perceptions of Everyday Objects" in exchange for $0.35 compensation. The mean age of the sample was 37.09 years (*SD*=12.85). The ethnicity of the sample was 90.9% White, 1% Hispanic/Latin American, 3% Black/African American, and 5.1% other or mixed ethnicities.

### Materials and Methods

The design and framing paralleled Study 2. Participants viewed images of adult male hands holding, respectively, a paintbrush, a squirt gun, and a kitchen knife, in that order (see Figure 2, Panel C). For each image, participants were asked to rate the harmfulness of each object, using a 9-point Likert scale (1=*Not at all dangerous*, 5=*Moderately dangerous*, 9=*Extremely dangerous*). For each image, participants were also asked which type of person would be most associated in their minds with the given object, with answers constrained to four fixed categories (adult women, adult men, young girls, or young boys). Participants were then asked demographic questions, thanked, and debriefed.

### Results and Discussion

#### Perceptions of Object Danger

A preliminary MANOVA revealed no effects of participant sex on ratings of object danger. Repeated measures ANOVAs with a Greenhouse-Geisser correction revealed a significant main effect of object condition for ratings of harmfulness, *F*(2.63, 257.74)=671.85, *p*<.000001, *η* ^2^_p_=.87. As predicted, planned contrasts revealed that the kitchen knife (*M*=7.65, *SD*=1.49) was rated as more dangerous than the paintbrush (*M*=1.63, *SD*=.86) or the squirt gun (*M*=2.42, *SD*=1.22).

#### Object Associations

Seventy-one percent of the sample rated the kitchen knife to be most associated with adult women, 92% of the sample associated the paintbrush with adult men, and 96% of the sample associated the squirt gun with young boys.

Having documented that kitchen knives are indeed associated with women, paintbrushes are indeed associated with men, and squirt guns are indeed associated with young boys, and having demonstrated that kitchen knives are indeed perceived as vastly more dangerous than paintbrushes or squirt guns, we then employed the same objects in a design directly paralleling that of Study 1 and Study 3.

## Study 5

### Participants

Six hundred and forty-seven participants living in the United States were recruited via the website MechanicalTurk.com to participate in exchange for $0.50 compensation. Screening using the same criteria employed in Studies 1 and 3 left a sample of 541 (336 female) with a mean age of 33.73 years (*SD*=12.1). The ethnicity of the sample was 80.2% White, 3.3% Hispanic/Latin American, 5.7% Asian, 6.5% Black/African American, and 4.3% other or mixed ethnicities.

### Materials and Methods

The design and framing paralleled Study 3. Three adult men having hands very similar in size and appearance served as models; the hand of each model was photographed holding a kitchen knife, a paintbrush, and a squirt gun. The photographs were then combined into three sequences such that, within a given sequence, each object was held by a different model, and each sequence presented the objects in a different order. Participants were again randomly assigned to view one of the three sequences of images.

### Results and Discussion

#### Effects of Object Order and Sex

MANOVAs revealed significant effects of participant sex on estimations of size and muscularity. As in Study 3, these effects were minor and inconsistent across objects; sex was therefore controlled for in subsequent analyses of these variables. A MANOVA also revealed significant effects of the order of image presentation on height, overall size, and muscularity estimates; once again, these effects were neither substantial nor patterned, and hence order of presentation was also controlled for in all subsequent analyses.

#### Height Estimates

Repeated measures ANCOVAs with a Greenhouse-Geisser correction controlling for order revealed a significant main effect of object condition for estimations of height, *F*(1.95, 1,048.93)=52.32, *p*<.000001, *η* ^2^_p_=.088 (see Table 4 for means). As predicted by our representation-of-formidability hypothesis, planned contrasts revealed that the men holding the kitchen knife were estimated to be taller than the men holding the paintbrush (*p*<.0001) and the squirt gun (*p*<.000001). Consistent with the notion that the squirt gun holders would be estimated as shortest due to a simple association with children, the men holding the squirt gun were estimated to be shorter than the men holding the paintbrush (*p*<.01).

**Table 4:** Estimated Height, Size, and Muscularity (Study 5).

	kitchen knife	paintbrush	squirt gun
height *Mean (SD)*	68.64 (2.51)	68.07 (3.03)	67.68 (3.03)
size *Mean (SD)*	3.18 (1.10)	3.05 (1.23)	2.81 (1.21)
muscularity *Mean (SD)*	2.35 (1.08)	2.17 (1.12)	2.13 (1.06)

*Note.* N=541. Estimated heights are in inches. The men whose hands were pictured holding the kitchen knife were estimated to be taller, larger, and more muscular than the other men (*p*s<.03). The men holding the squirt gun were estimated to be shorter and smaller than the men holding the paintbrush (*p*s<.01); see text for analyses.

#### Size Estimates

A repeated measures ANCOVA controlling for order of object presentation and sex of participant revealed a significant main effect of object condition for estimations of size, *F*(1.93, 938.59)=9.21, *p*<.001, *η* ^2^_p_=.02 (see Table 4 for means). As predicted, planned contrasts revealed that the men holding the kitchen knife were estimated to be larger than the men holding the paintbrush (*p*<.03) or the squirt gun (*p*<.000001). Consonant with the height estimates, the men holding the squirt gun were estimated to be smaller than the men holding the paintbrush (*p*<.01).

#### Muscularity Estimates

A repeated measures ANCOVA with a Greenhouse-Geisser correction controlling for order of object presentation and sex revealed a main effect of object condition for estimations of muscularity, *F*(1.96, 1,045.19)=21.79, *p*<.000001, *η* ^2^_p_=.039 (see Table 4 for means). As predicted, planned contrasts revealed that the men holding the kitchen knife were estimated to be more muscular than the men holding the paintbrush (*p*<.01) or the squirt gun (*p*<.0001). Breaking with the height and size estimations, the men holding the squirt gun and those holding the paintbrush were not estimated to differ from one another in muscularity (*p*>.45).

In Study 5, participants estimated men to be larger and stronger when the target individual possessed a kitchen knife, an object principally associated with women, and one that does not require strength to use effectively. This finding indicates that the core results of Studies 1 and 3 are not primarily explicable in terms of cultural schemas involving associations between guns and large, muscular men, nor are they explicable in terms of associations stemming from the strength needed to properly handle a large handgun. Participants in Study 5 did estimate men to be shorter and smaller when the target possessed a toy squirt gun, an object associated with children, thus indicating that schematic cultural associations between objects and categories of persons likely do play some role in estimations of this type. Compellingly, however, comparison of the effects of the kitchen knife (associated with women) with those of the paintbrush (associated with men) indicate that, when lethal affordances characterize an object that is associated with a comparatively shorter, smaller, and physically weaker class of individuals, the impact of said lethal affordances overrides any influence of schematic associations, leading to a net magnification of estimations of the size and strength of the object's possessor.

## Discussion

Knowing that an individual possesses a potentially lethal object, be it a handgun or a kitchen knife, led our U.S. participants to generally conceptualize the target individual as taller and larger in overall body size and muscularity. Our auxiliary investigations indicate that these patterns are not explicable in terms of cultural schemas linking bodily properties to the objects at issue, nor can they be explained in terms of background knowledge regarding the actual properties of gun owners. These findings constitute preliminary evidence in support of the hypothesis that conceptualized size and strength act as key dimensions in a cognitive representation that summarizes the formidability of a potential foe, where possession of a weapon is one factor contributing to said formidability.

As discussed in the Introduction, both phylogenetic and ontogenetic considerations predict the existence of the hypothesized representational system. The phylogenetic thesis and the ontogenetic thesis are mutually compatible, as experiences that predictably occur during ontogeny often serve as the avenue whereby evolved adaptations develop. However, it is also possible that either a) the postulated representational system is entirely innate, and hence is independent of experience, or b) the postulated representational system is entirely the product of experience processed by a domain-general learning system, and hence does not reflect a discrete evolved adaptation. Given the positive affordances for solving adaptive problems provided by the prolonged period of human maturation (see, for example, [10]), we think that (a) is unlikely. Likewise, because the crucial adaptive problem of assessing relative formidability vastly predates mammalian sociality and altriciality, we think that (b) is also unlikely. We therefore favor a hybrid thesis that postulates the existence of an evolved adaptation, the successful functioning of which is at least partially contingent on predictably recurrent experiences during development. Nevertheless, we recognize that the results presented here are compatible with all three of these possibilities. Lastly, although both the phylogenetic thesis and the ontogenetic thesis logically apply to many social species, as we have investigated this hypothesis only in humans, applications to other species remain speculative, awaiting the development of experimental methods for assessing size estimation in nonhumans.

Prior work in humans indicates that information regarding an individual's social status also influences perceptions of the individual's size (reviewed in [11]; see also [12] and [13]). Recently, Marsh, Yu, Schechter, and Blair [14] demonstrated that nonverbal cues associated with social status exercise a similar influence. In humans, status can reflect either dominance (i.e., position achieved through force or the threat thereof), prestige (i.e., position achieved through deference freely granted by others in light of accomplishments), or a combination of these factors [15] and [16]. While dominance is a universal feature of status hierarchies in social animals, prestige is thought to be unique to humans [15] and [16]. This suggests that the psychological mechanisms with which humans navigate status hierarchies initially evolved to address dominance, and were subsequently modified in our lineage to also address prestige (cf. [17]). We can therefore expect that the human representational systems that address status, having evolved from systems concerned with formidability, likely employ physical size to summarize diverse factors affecting social position.

Viewed in this light, the aforementioned existing findings likely accurately capture the manner in which status is conceptually represented in humans. However, while we find this account compelling, we also recognize that previous findings linking status and perceived size may also owe to an alternative explanation. Height is correlated with actual social position and corresponding social influence – taller people achieve greater professional success, are paid more, are more likely to be elected, and so on (reviewed in [13] and [14]). Accordingly, participants may perceive high-status individuals as taller simply due to prior knowledge regarding the correlation between height and status. Likewise, cues of social superiority may lead to increases in perceived size [14] because participants may know that taller people often occupy elevated positions in the social hierarchy, and hence indications of high rank may lead to inferences of above-average height. Importantly, however, Duguid and Goncalo [18] have recently presented evidence that both militates against such an inferential explanation and is consonant with our thesis that relative formidability is represented in part using the dimension of size. In an elegant series of studies, the authors demonstrate that manipulating participants' perceptions of their ability to exercise power over others (either via unspecified means, or via a managerial position in a corporation) leads participants to a) increase estimates of their own physical height, b) decrease estimates of another person's physical height, and c) increase the height of a computerized avatar selected to represent themselves.

Our findings are not readily explained in terms of participants' inferences derived from their prior observations of simple associations in the world, as gun owners are not taller than non-owners, and kitchen knives are associated with women, yet knowing that a man possesses a gun or a kitchen knife leads people to assess him as larger and more muscular. In conjunction with prior work, our studies thus provide strong preliminary evidence that the conceptual dimensions of size and strength are employed to represent relative formidability. In the future, we aim to arrive at similar clarity regarding the direction of causality in the relationship between overall social status and perceptions of size, a key step in exploring the evolution of the psychology of social hierarchy in humans.
